# Characterization of the complete chloroplast genome of *Camellia yuhsienensis* Hu, a resilient shrub with strong floral fragrance

**DOI:** 10.1080/23802359.2020.1797580

**Published:** 2020-07-26

**Authors:** Ziyan Nie, Xingzhao Huang, Zhikang Hu, Xinlei Li, Hengfu Yin, Jiyuan Li

**Affiliations:** a School of Forestry and Landscape Architecture, Anhui Agricultural University, Hefei, China; b Key Laboratory of Forest Genetics and Breeding, Research Institute of Subtropical Forestry, Chinese Academy of Forestry, Hangzhou, China

**Keywords:** *Camellia yuhsienensis* Hu, chloroplast genome, phylogenetic analysis

## Abstract

*Camellia yuhsienensis* Hu is an economically valuable species in the genus *Camellia*. It is widely used for breeding ornaments and oil varieties. In this study, the complete chloroplast (cp) genome sequence of *C. yuhsienensis* is assembled and annotated. The whole cp genome of *C. yuhsienensis* is 156,912 bp in size, composed of a small single copy (SSC) region of 18,296 bp and a large single copy (LSC) region of 86,560 bp, separated by a pair of inverted repeats (IRs, IRA: 86,561–112,588; IRB: 130,885–156,912). The overall GC content of *C. yuhsienensis* cp genome is 37.3%, with the base content A (31.08%), T (31.63%), C (19.02%), and G (18.27%). The phylogenetic analysis of 15 *Camellia* species based on 77 protein-coding genes shows that *C. yuhsienensis* is evolutionarily close to *Camellia taliensis*.

*Camellia yuhsienensis* Hu is an excellent germplasm resource due to its strong floral fragrance, high oil content and high resistance to anthracnose (Kuang et al. 2015). It is initially discovered in Youxian area (Hunan Province, China) and is widely used for breeding varieties of ornaments and oil. As a wild relative of *Camellia oleifera* (commonly known as ‘Oil-Camellia’), *C. yuhsienensis* has a compact tree shape and bears white fragrant flowers in spring. The chloroplast (cp) genome is featured with high sequence conservation and has great value in revealing the origin, evolution, and kinship of different species (Allen. 2015). It has been extensively used to obtain knowledge of phylogeny and genetic diversity (Li et al. 2019). However, the genomic information of *C. yuhsienensis* is limited. Here, we sequenced the cp genome of *C. yuhsienensis* through the high-throughput sequencing and described the assembly and annotation (NCBI GenBank Accession Number: MT665973). We further analyzed the phylogenetic relationship with its closely related species. This study provides an important sequence reference for its future biological research.

The research materials (voucher number: CY1) are reserved in State Key Laboratory of Tree Genetics and Breeding, Research Institute of Subtropical Forestry, Chinese Academy of Forestry (Hangzhou City, Zhejiang Province, China), Coordinates: 29°44′45″–30°11′58.5″N, 119°25′–120°19.5′E. The high-quality DNA was obtained by using a TruSeq DNA sample preparation kit (Vanzyme, CHN), and tested using a NanoDrop 2000 device (Thermo Fisher Scientific, USA) as described (Sarnecka et al. 2019). The fragment peaked at 300 bp was used to construct a sequence library by Agencourt AMPure XP-PCR Purification Beads (Beckman Coulter, USA) and Agencourt SPRIselect (Beckman Coulter, USA). After quality checking, the library was sequenced using Illumina Hiseq 2500 sequencing systems (Illumina, USA) at Genesky Biotechnologies (Shanghai, China). Finally, the cp genome map of *C. yuhsienensis* was generated using OGDRAW version 1.3.1 (Greiner et al. 2019).

We gained 24,743,671 reads and 3,582,740,603 bases after quality control using Trimmomatic (Bolger et al. 2014). We had assembled the original data of cp genome of *C. yuhsienensis* using the MetaSPAdes (Wang et al. 2018). The annotation of the genome was performed using the program OrganellarGenomeDRAW (Lohse et al. 2013), and then manually confirmed through comparing with the cp genome of *C. yuhsienensis*. The final assembly complete cp genome sequence of *C. yuhsienensis* is 156,912 bp with the typical quadripartite structure. It is constituted by an LSC region of 86,560 bp, and SSC region of 18,296 bp and two pairs of IRs region(IRs, IRA: 86,561–112,588 IRB: 130,885–156,912). The total GC content of the sequence was 37.3%. The cp genome of *C. yuhsienensis* has 130 functional genes, which are composed of 77 protein-coding genes, 43 tRNA genes, and four rRNA genes. We identified 77 protein-coding genes that were conserved between 15 *Camellia* species to construct the phylogenetic relationships using the perl script (Wang et al. 2019). As shown in Figure 1, *C. yuhsienensis* and *Camellia taliensis* have the closest relationship.

**Figure 1.**
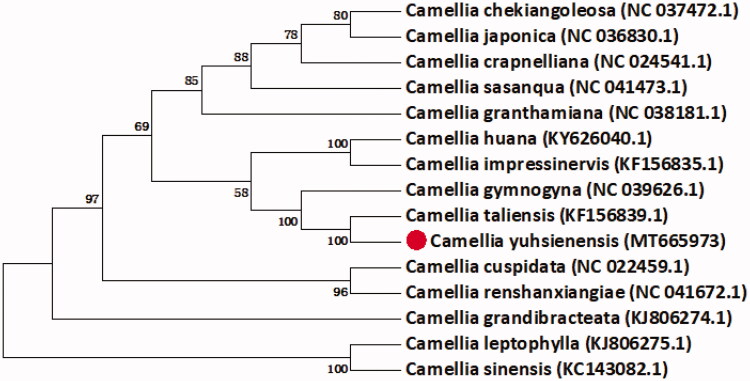
The neighbour-joining phylogenetic tree of 15 *Camellia* cp genomes was conducted with MEGA v7.0.14 (Sudhir et al. 2016). The bootstrap values from 100 replicates are listed for each node.

## Data Availability

The data that support the findings of this study are openly available in GenBank of NCBI at https://www.ncbi.nlm.nih.gov, reference number MT665973, or available from the corresponding author.
